# FedVoting: A Cross-Silo Boosting Tree Construction Method for Privacy-Preserving Long-Term Human Mobility Prediction

**DOI:** 10.3390/s21248282

**Published:** 2021-12-10

**Authors:** Yinghao Liu, Zipei Fan, Xuan Song, Ryosuke Shibasaki

**Affiliations:** 1 Center of Spatial Information Sciences, The University of Tokyo, Kashiwanoha 5 Chome-1-5, Kashiwa 277-0882, Japan; yinghaoliu@g.ecc.u-tokyo.ac.jp (Y.L.); fanzipei@iis.u-tokyo.ac.jp (Z.F.); shiba@csis.u-tokyo.ac.jp (R.S.); 2 SUSTech-UTokyo Joint Research Center on Super Smart City, Department of Computer Science and Engineering, Southern University of Science and Technology (SUSTech), Shenzhen 518055, China

**Keywords:** long-term human mobility prediction, privacy protection, federated learning, gradient-boosting decision tree, differential privacy, GPS

## Abstract

The prediction of human mobility can facilitate resolving many kinds of urban problems, such as reducing traffic congestion, and promote commercial activities, such as targeted advertising. However, the requisite personal GPS data face privacy issues. Related organizations can only collect limited data and they experience difficulties in sharing them. These data are in “isolated islands” and cannot collectively contribute to improving the performance of applications. Thus, the method of federated learning (FL) can be adopted, in which multiple entities collaborate to train a collective model with their raw data stored locally and, therefore, not exchanged or transferred. However, to predict long-term human mobility, the performance and practicality would be impaired if only some models were simply combined with FL, due to the irregularity and complexity of long-term mobility data. Therefore, we explored the optimized construction method based on the high-efficient gradient-boosting decision tree (GBDT) model with FL and propose the novel federated voting (FedVoting) mechanism, which aggregates the ensemble of differential privacy (DP)-protected GBDTs by the multiple training, cross-validation and voting processes to generate the optimal model and can achieve both good performance and privacy protection. The experiments show the great accuracy in long-term predictions of special event attendance and point-of-interest visits. Compared with training the model independently for each silo (organization) and state-of-art baselines, the FedVoting method achieves a significant accuracy improvement, almost comparable to the centralized training, at a negligible expense of privacy exposure.

## 1. Introduction

Human mobility has a huge impact on urban management and business activities. The prediction of human mobility can estimate people flows at points of interest (POIs) in advance based on the history of mobility data. Therefore, it can facilitate solving many kinds of urban problems, such as traffic congestion, air pollution, epidemic spread, etc. In commercial activities, human mobility is also fundamental for location-based services (LBSs). Based on the prediction of human mobility, companies can acquire the future location of users and supply more accurate services, such as advertisements and route recommendations.

With the widespread use of smartphones and portable devices, a substantial amount of valuable personal data (such as GPS trajectories) is generated every day, which could be put together to improve the performance of human mobility prediction. However, to prevent privacy leakage and private information abuse, the protection of personal data is becoming a global concern. In 2018, the European Union enforced the General Data Protection Regulation (GDPR) to protect individual privacy, which sets many constrains on companies’ usage of personal data. Thus, relevant companies, such as map service providers, can collect very limited data; it is also hard for them to share these data with other companies. These data are distributed in “isolated islands”, making it less possible to utilize them efficiently. In this context, federated learning (FL) architecture is a very suitable method for using private data in different entities to achieve a better performance of applications in a privacy-preserving way. Federated learning is a machine learning technique that many entities (also referred to as silos, when the participants are organizations) collaboratively train a model under the orchestration of a central server [1,2]. The core characteristic is that each entity’s raw data are stored locally and not exchanged or transferred in the whole process. Therefore, data privacy can be effectively protected without uploading raw data to the central server, which happens in traditional machine learning methods and raises privacy concerns. By combining federated learning, the privacy issues in human mobility prediction can be resolved.

In terms of the prediction period, long-term human mobility prediction is an important but challenging research direction. Compared with short-term prediction (where people would go in the next hours), long-term human mobility prediction usually focuses on a longer period (where people would go in the next weeks or months), which can supply a more sufficient response period for government or companies to take correlated measurements, such as security strengthening or advertising. However, long-term human mobility is more difficult to predict due to the complexity of mobility patterns over a longer period. Generally, human mobility patterns include regular patterns (such as commuting on weekdays) and irregular patterns (such as activities on weekends). Long-term human mobility consists of more irregular patterns in addition to people’s daily routines. For example, whether or not people would be attending special events or visiting specific POIs in weeks or months, the mobility of which is irregular and where attendance is infrequent. Therefore, it is an interesting topic to be further explored. Many research studies have mainly focused on short-term human mobility prediction. For example, Ref. [3] proposed a model called CityMomentum to predict human movements in the next hour based on a clustering framework and a mixture of multiple random Markov chains. The authors of [4] also supplied a model (DeepMove) to predict short-term mobility based on attentional recurrent networks. Short-term human mobility prediction can only function on small regions due to the limit of mobility speed; further, it cannot supply sufficient response time in practical applications.

Thus, how can we effectively tackle the irregularity-related problems of long-term mobility? In addition to extracting richer features from long-term trajectory data, the selection of the base model is also crucial. The gradient-boosting decision tree (GBDT) model is an ensemble model which trains a sequence of decision trees and uses these weak trees to constitute strong decision trees in a boosted way. The GBDT model is a high-performance machine learning model and won many awards in machine learning and data mining competitions [5]. The tree structure of the GBDT can tackle high-dimensional data very well and is suitable for the complex features extracted from long-term mobility data. Combined with the architecture of federated learning, the optimized GBDT model can also achieve high performance on the premise of privacy protection. There have been some works on the GBDT model with federated learning for general datasets. The authors of [6] designed a privacy-preserving scheme for the GBDT model, where individual data owners can perform training locally based on differential privacy (DP); then, different trees trained by multiple data owners can be securely aggregated into an ensemble. Federated extreme gradient-boosting (FEDXGB) realized a federated GBDT structure where participants upload their gradients and the central server serves as the training role by utilizing the secure aggregation scheme based on secret sharing and homomorphic encryption (HE) [7]. The authors of [8] introduced a similarity-based federated learning structure where each participant boosts a number of trees by exploiting gradients of similar instances from other participants; then, the trees generated by all participants can form an ensemble GBDT model.

However, these state-of-art privacy-preserving GBDT models have some weaknesses. In [6], both the splitting nodes and the leaf nodes are added the same-level noises with differential privacy, which severely deteriorates the model’s performance. Moreover, the ensembles of GBDTs are trained in a fixed sequence of participants. The model’s performance is also impaired in conditions of non-balanced distribution of data volume or data quality among participants. Therefore, we aim to design a kind of heterogeneous method based on differential privacy to separately protect the splitting and leaf nodes; furthermore, the model can be trained flexibly in each round to achieve the best performance. Additionally, in [7,8], participants need to transmit the gradients of their local data to the server or each other. The gradients are mapped or queried from the original data, which are very sensitive and can be inferred or deciphered by malicious or semi-honest entities leading to privacy leakage. Although [7] applied homomorphic encryption and [8] utilized local-sensitivity-hashing to serve as the protective measurements, these procedures lead to the increase in computation complexity and communication costs. Therefore, we argue that the GBDT model can be trained completely in the local silo to prevent raw data and gradients from transferring, which can effectively avoid privacy leakage. By designing an optimized construction method for the GBDT ensemble, long-term human mobility prediction can be achieved with both good performance and privacy protection.

Specifically, the ensemble of GBDTs consists of decision trees in a boosted way, and, when training, the new decision trees are added into the ensemble one by one, which means that the GBDT ensemble can be constructed in a flexible way; namely, different participants (silos) can train the decision trees locally on their raw data, and then upload these decision trees to the server and aggregate them together to generate the final ensemble. In this way, the silos just need to upload the parameters of the trained trees instead of more sensitive raw data or gradients. Furthermore, the tree parameters can be protected by differential privacy (DP) based on the addition of noises. By the specific analysis of the range and scale of added noises, a balance between performance and privacy protection can be achieved. More importantly, if the decision trees are just trained by sequenced silos, the performance of the final ensemble of GBDT would be impaired due to the varied data conditions of the silos. Therefore, we designed an optimized method to choose the best trees trained by different silos in each iteration to construct the optimal ensemble of the GBDT model.

**Figure 1:**
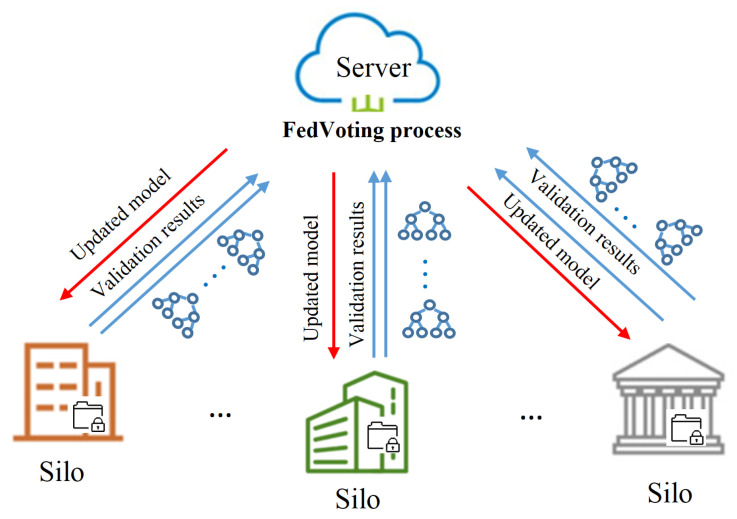
The architecture of the cross-silo federated GBDT model.

For long-term human mobility prediction, we propose a novel and practical federated GBDT architecture with the constructing method called FedVoting, where trees are trained locally by each silo, then cross-validated by other silos with their raw data and, in the final voting step, the validation results serve as the votes to decide the optimal trees in the current iteration. After multiple rounds of iterations, the optimal ensemble of GBDTs is implemented with the voted trees in each iteration. As Figure 1 shows, the participated silos just need to upload their model parameters and validation results to the server, while their raw data can be kept locally without any uploading or transferring. The central server (or one of the silos) just acts as the operation controller to conduct the FedVoting process and update the voted model to all silos, without acquiring sensitive information.

In summary, we achieved the following key contributions:

(1) We propose a novel federated voting method to construct GBDT ensembles that facilitates data owners in collaboratively training a more accurate model than a model trained independently, without the risk of data leakage, in a concise and practical way.

(2) We designed a heterogeneous method to further protect the parameters of the decision trees based on differential privacy. By adding different scales of noises separately to splitting nodes and leaf nodes, an optimized compromise between privacy protection and performance can be achieved.

(3) We analyze two cases of long-term human mobility prediction (including special event attendance and POI visits) based on real-world GPS data. The experimental results show that the federated GBDT model trained by the FedVoting process achieved excellent accuracy close to the original non-federated GBDT method, demonstrating a significant improvement compared with the state-of-art baseline, as well as in respect to independent training performed by each participant. We also propose the motivation mechanisms to attract participants based on the quantified contributions.

## 2. Related Work

**Urban computing:** Urban computing has a very wide research scope, aiming to address specific problems in urban life by utilizing different kinds of data, such as GPS data [9,10,11], Wi-Fi and Bluetooth data [12,13], social network data [14], crowd-sourcing temperature and humidity data [15], etc. We also focus on solving urban problems mainly based on GPS data, which can show more accurate information of the locations and trajectories of people’s daily activities than other data sources. With big GPS data, rich patterns can be extracted to make future predictions. Under the aspect of activity attendance prediction, Ref. [16] proposed a singular value decomposition with multi-factor neighborhood (SVD-MFN) algorithm to predict activity attendance by integrating the data sources of social network services (SNS). The authors of [17] introduced a kind of attendance prediction method for both outdoor and indoor activities based on weather data and the gradient-boosting tree model. Regarding POIs, Ref. [18] proposed a POI recommendation approach to help users make travel plans by utilizing the data collected from location-based social networks (LBSNs). The aim of the authors of [19] was to predict potential visitors for a given POI by designing a method to jointly model user preference and POI sequential transition influence. In this research study, we integrate activity attendance prediction and POI visit protection in one mobility problem, to achieve a more accurate prediction by extracting rich features from big fine-grained GPS data.

**Human mobility prediction:** Human mobility has huge impacts on urban areas under many aspects and is an attractive research field. There have been many researchers that have explored problems related to human mobility prediction [20,21,22]. In addition, Ref. [23] built the online system called DeepUrbanMomentum to conduct short-term mobility prediction by using recurrent neural networks (RNNs) with currently observed human mobility data. The authors of [24] proposed a kind of attention-based human mobility predictor for short-term human mobility prediction, which can be trained and predicted in a decentralized way without collecting user data in the server. The authors of [25] constructed a hybrid Markov-based model considering the non-Gaussian and spatio-temporal characteristics of real human mobility data to predict people’s future movements. However, these works mainly designed network models exhibiting temporal dynamic behavior to predict short-term human mobility, e.g., in next hour. Long-term trajectories would show significantly different patterns, such as commuting patterns on weekdays and irregular mobility on weekends, which are also meaningful and can be extracted for solving urban problems, such as the extracted travel-frequency patterns in this research study. We abstracted the long-term prediction into a classification problem and designed the novel model-construction method for prediction in a privacy-preserving way.

**Federated learning:** For the privacy protection of big data, there are a number of privacy-preserving mechanisms in the life cycle of big data [26], such as k-anonymity in data generation [27] and blockchain in data storage and management [28]. In data processing and applications, there has been increasing research interest in traditional machine learning models combined with the federated learning method to protect user privacy or avoid data leakage, meanwhile attaining or remaining close to the original performance [29,30,31]. Moreover, FedMA utilizes the federated-matched averaging algorithm to combine federated learning with deep convolutional neural networks (DCNN) and long short-term memory networks (LSTM), achieving good performance with real-world datasets in terms of privacy protection [32]. The authors of [33] focused on image datasets and implemented two mainstream object detection algorithms (YOLO and Faster R-CNN) in federated learning scenarios. The authors of [34] proposed a federated transfer learning (FTL) model to enhance the efficiency and security of existing models for collaborative training under data federation by incorporating secret sharing (SS). Although these federated models constitute various effective models in a privacy-preserving way, they do not shed light on the scenarios of human mobility prediction with irregular trajectory data. Furthermore, Ref. [35] proposed a privacy-preserving mobility prediction framework called PMF based on the long short-term memory (LSTM) model and federated learning method to predict users’ next location. The authors of [36] proposed a personalized federated learning model named AMF (adaptive model-fusion federated learning) with a mixture of local model STSANs (spatial-temporal self-attention networks) and global model to predict users’ next location. However, these models only function on short-term mobility prediction by utilizing the sequential characteristics of mobility data. In this research study, the federated GBDT model is constructed by the proposed FedVoting mechanism via the extraction of high-dimensional features from long-term irregular GPS trajectory data. Therefore, long-term human mobility (e.g., one month) can be accurately predicted in a privacy-preserving way and the compromise between performance and privacy protection can be effectively achieved by the designed heterogeneous differential-privacy method.

## 3. Preliminaries

There are two main settings related to the participants in federated learning, i.e., cross-silo and cross-device. Cross-silo federated learning means the participants are organizations (e.g., companies and data centers) with large volumes of data, while cross-device federated learning consists of a very large number of mobile devices or IoT terminals with fewer data [1]. We focus on the cross-silo setting because organizations hold the human mobility data of their users and desire to acquire a more accurate model to predict the long-term mobility of their users to supply better services. Cooperation to train a collective model is an optimal choice among organizations. However, they cannot put the data together or share the data with others due to privacy issues. Therefore, participating in federated learning is a good pathway for both high-performance models and data privacy protection. The features of the data in all the silos (we use silos to represent the participants in cross-silo FL) are the same. Thus, it can also be classified as a horizontal federated-learning problem [2].

The human mobility prediction in this research study is based on long-term GPS trajectory data. Raw GPS data are pre-processed to the travel-frequency patterns and then serve as the features for prediction. To clearly demonstrate these methods, in this section, we define the terms and concepts frequently used through this paper.

**Definition** **1** (Raw GPS data)**.** *GPS data is a kind of data format for human mobility recording with relatively higher precision than check-in data or call detail records (CDRs); GPS data also have a larger sampling frequency to generate fine-grained trajectories. In the trajectory history of a user, GPS data contain many kinds of elements, among which timestamp, latitude and longitude, consisting of a 3-tuple, are commonly utilized and can be formally represented as follows:*

(1) X={(t,lat,lon)}

*where X represents one record of GPS data format. Then, the trajectory in the form of GPS for the user u can be defined as follows:*

(2) $$Traj_{u}=\left\{x_{u,i}|x_{u,i}\in X_{u},i\in N\right\}$$

*where $x_{u,i}$ represents the i-th GPS record in a temporal sequence.*

**Figure 2:**
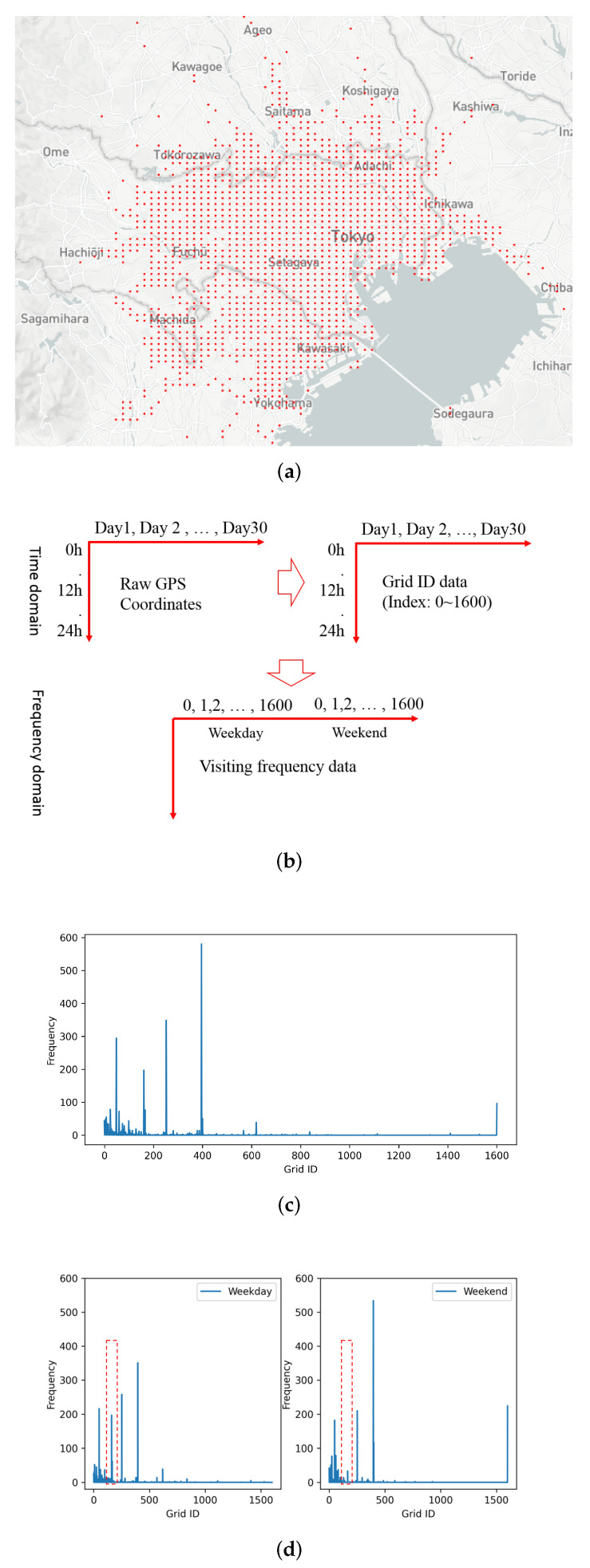
Illustration of travel-frequency pattern preprocessing. (**a**) Grid distribution. (**b**) The procedures of preprocessing. (**c**) Travel pattern without distinguishing weekday from weekends. (**d**) Travel pattern distinguishing weekdays from weekends.

**Definition** **2** (Grid-level trajectory)**.** *In the prediction of long-term human mobility, the destinations are POIs or places for holding special events, which can be represented by grids (square area with the same size). In the same way, the GPS coordinates in the trajectory can also be transformed into grids for further processing. As grid information can show the characteristics of long-term mobility in the frequency domain, it can facilitate the extraction of useful features. Moreover, in the aspect of privacy protection, the grid-level trajectory is more coarse and less sensitive than the temporal GPS trajectories with timestamps. We can formalize the locations in the grid as $D=\{d_{i}=\left(lat_{i},lon_{i}\right)|i\in \left(1,M\right)\}$, where $d_{i}$ represents the grid id of the location, $\left(lat_{i},lon_{i}\right)$ is the center of each grid with a fixed scale and M is the number of grids in the research area (refer to Figure 2a). Thus, the GPS coordinates in the user trajectory can be matched to the nearest grids. Further, the trajectory can be represented by the sequence of grids.*

(3) $$Traj_{u}=\left\{d_{u,i}|d_{u,i}\in D,i\in \left(0,L-1\right)\right\}$$

*where L is the length of trajectory and i is the ith grid in the trajectory. To make the trajectory more uniformly distributed, we conduct the interpolation for the trajectories to generate the grids in a fixed time interval—for example, 15 min.*

**Definition** **3** (Travel-frequency pattern)**.** *To better extract the trajectory features of the user, we introduce the travel-frequency pattern to describe the characteristics of the user’s mobility in a long period, such as weeks or months. Specifically, the travel-frequency pattern represents the visiting frequency in the pre-defined grids at a fixed period. When there are enough grids, the features of a travel pattern can be fully extracted in the wide-distributed grids. The travel-frequency pattern can be formalized as follows:*

(4) $$S_{u}^{T}=\left\{f_{u,d_{i}}^{T}|d_{i}\in D,i\in \left(1,M\right)\right\}$$

*where $S_{u}^{T}$ represents the travel-frequency pattern of user u in the period T and $f_{u,d_{i}}^{T}$ means the visiting frequency of the user u at grid $d_{i}$ in the period T.*

**Definition** **4** (Long-term human mobility prediction)**.** *In this study, we focus on the long-term prediction of human mobility. Specifically, we predict whether the users would attend the special event or visit the point of interest (POI) in the next month based on the whole current month’s trajectory data (T=onemonth), which can be formalized as $p(y_{u}|S_{u}^{T})$, where $y_{u}$ is a binary classification, with 0—no; 1—yes.*

## 4. Travel-Frequency Pattern PRE-PROCESSING

In this section, we introduce the procedures of preprocessing where the raw GPS data of a fixed period (commonly one month) are finally transformed into visiting frequency data among grids, namely, the travel-frequency pattern of users, which serve as the features to predict long-term human mobility. Specifically, we take the Great Tokyo Area as the research region and generate the grid data based on the standard grid square and grid square code [37]. Through the statistics of human mobility data, the most frequently visited 1600 grids are chosen as the research objects. The distribution of interested grids are shown in Figure 2a, where the red points represent the center of grids.

The procedures of preprocessing are shown in Figure 2b. Firstly, the raw GPS data are clustered to the nearest grids, which can transform the trajectory from the GPS format into grid ID. In this process, forward-interpolating is utilized to generate the uniformly distributed grid sequence. Commonly, the GPS trajectory is sampled in a fixed-time interval, such as 15 min. However, if there are no trajectory records in the next 15 min, forward-interpolating sets the current grid ID as the grid ID of the next 15 min. In this way, the trajectory in grid format is temporally continuous and complete for conducting further processing.

Next, the grid-level trajectory is transformed from the time domain into the frequency domain, by which the travel-frequency pattern can be extracted. Specifically, for a user in the given period (one month), the visiting frequencies of each grid in the research area are counted and summarized. Then, we can obtain the one-month travel-frequency pattern of this user (shown in Figure 2c), which can provide the features to conduct the prediction of the user’s mobility in next month.

To extract more features from the long-term trajectories, we conduct further analyses. As shown in Figure 2c, several grids are more frequently visited than others, which are normally the user’s home, work place and shopping or eating premises. Moreover, commonly, the patterns of frequently visited places on weekdays are different from weekends for the same user. On weekdays, the commuting pattern for workers dominates and work places are more frequently visited, such as the grid in the red-dotted frame in the left graph in Figure 2d. Conversely, on weekends, the grids of work places are less visited. Therefore, to more accurately capture the features of travel-frequency patterns, we divide the total month into weekdays and weekends and separately count the visiting frequency of all grids; then, we concatenate these two groups to generate the final travel-frequency patterns. In this way, the travel-frequency patterns are in a higher dimension with richer features.

## 5. Long-Term Human Mobility Prediction

This section introduces the specific implementations of the proposed FedVoting process to construct federated GBDTs for long-term human mobility prediction and includes three subsections. In the first subsection, “Base model selection”, we show the strengths of selecting the GBDT model as the base model and introduce the characteristics of the boosting training method. In the design of federated learning, even though we keep the raw data and gradients in local positions, the parameters of the model are still uploaded or transferred, which implies the risk of privacy leakage. Therefore, in the second section, “Heterogeneous privacy protection with differential privacy (DP)”, we introduce the designed heterogeneous method based on differential privacy to separately protect the parameter of leaf nodes and splitting nodes in the GBDT ensemble. Finally, in the last subsection, “FedVoting process to construct Federated GBDT model”, the core idea and specific implementation processes are introduced in a detailed way.

### 5.1. Base Model Selection

Combined with the architecture of federated learning, the selection of a specific base model is crucial for the performance of long-term human mobility prediction. Considering the complexity and irregularity of long-term mobility, we extract higher-dimensional features from the trajectory histories, called travel-frequency patterns. Therefore, the base model needs to have a good capacity to tackle high-dimensional data and also needs to be able to integrate efficiently with federated learning to protect data privacy.

There are many popular models that are powerful in processing high-dimensional data, such as the gradient-boosting decision tree (GBDT) and random forest models, which are based on tree models, and support vector machine (SVM), multilayer perception (MLP), etc. Another perspective is to decrease the complexity of the features when facing high-dimensional data, such as in the case of the factorization machine (FM). However, for long-term human mobility data, one instance represents one user, while the silos, commonly, just have limited users. Therefore, the decision tree-based models are preferred due to their strengths on datasets with small-volume instances. To quantitatively compare the effectiveness of these models on travel-frequency patterns, we tested the prediction accuracy of long-term human mobility on real-world GPS data. As shown in Table 1, the GBDT model had a higher accuracy than other models, which shows that GBDT is the optimal base model.

GBDTs consist of a sequence of boosted decision trees. A decision tree contains multi-layer nodes with optimal splitting values of chosen features, which can achieve a better performance in the dataset with high-dimensional features. Given a dataset $\left\{\left(x_{i},y_{i}\right)\right\}$, the GBDT model can be formalized as ${\hat{y}}_{i}=\sum_{k=1}^{t}f_{k}\left(x_{i}\right)$, where ${\hat{y}}_{i}$ is the final prediction result, $f_{k}\left(x_{i}\right)$ is the prediction result of the *k*-th tree and *t* is the number of total trees. Thus, the final prediction result of the GBDT model is the sum of each tree’s results.

GBDTs are trained in an additive way, with the decision trees being added into the ensembles one by one, which is the foundation of the proposed FedVoting process introduced later in the paper. This training method can be formalized as ${\hat{y}}_{i}^{\left(t\right)}={\hat{y}}_{i}^{\left(t-1\right)}+f_{t}\left(x_{i}\right)$, where ${{\hat{y}}^{\left(t-1\right)}}_{i}$ is the prediction result of previously generated (t−1) trees and $f_{t}\left(x_{i}\right)$ is the *t*-th tree to be trained. The goal of training a tree is to minimize the loss of objective function by selecting the splitting point. Formula (5) shows the objective function $L^{\left(t\right)}$:

(5) $$L^{\left(t\right)}=\sum_{i=1}^{n}l\left(y_{i},{\hat{y}}_{i}^{\left(t\right)}\right)+\sum_{i=1}^{t}\Omega \left(f_{i}\right)$$

where *i* is the index of each sample, *n* is the total number of training samples in the dataset and $y_{i}$ is the label of the *i*-th sample; $l(y_{i},{\hat{y}}_{i}^{\left(t\right)})$ represents the loss between the ground truth and the predicted label in the *t*-th iteration and $\Omega (f_{i})$ is a regularization term to penalize the complexity of the tree.

### 5.2. Heterogeneous Privacy Protection with Differential Privacy (DP)

Human mobility data are valuable and can be utilized to advance the performance of applications. Therefore, all silos desire to acquire or infer more information about the mobility data from other silos in federated learning. Moreover, these data belong to individual privacy. Although the silos can hold the data of their users, they have difficulties in sharing them due to the regulations. To decrease the leakage of individuals’ privacy, the training processes of the GBDT model are conducted locally on participated silos and just the trained model is uploaded to the server. Data privacy is naturally protected, without uploading or transferring any raw data. However, in extreme conditions, the trained model that each silo uploads or transfers is still at risk of exposing the original data [6,38]. This is because the threshold values in the decision trees are related to the features of the original data, while the leaf nodes are derived from the labels of the original data. We assume that the participants are curious and semi-honest. The single participant (including the server) or the collusion of participants all try to decipher more information from the models trained by other participants.

Therefore, we need to protect the parameters of the decision trees trained in local silos before any transferring or uploading happens. Differential privacy (DP) is an effective tool to protect the tree parameters and compromise between model accuracy and risk of privacy leakage. The core idea of differential privacy is to add random noises locally to sensitive data. After uploading or transferring the noise-added data, the original information is hard to be inferred or obtained. Therefore, to control the scale of added noises for the trade-off of performance and privacy protection, we apply the ϵ-differential privacy mechanisms. ϵ is a positive real number used to indicate the magnitude of the noises added into the dataset [39]. The noises are generated by randomized algorithms, such as the Laplace distribution, exponential distribution, etc. We use A(x) to denote the randomized algorithm; then, the ϵ-differential privacy can be shown as below:

(6) $$Pr\left[A\left(D_{1}\right)\in S\right]\leq exp\left(\epsilon \right)*Pr\left[A\left(D_{2}\right)\in S\right]$$

where Pr[.] means the probability of related functions, $D_{1}$ and $D_{2}$ refer to the datasets that differ by a single element in the dataset and *S* means the set-related dataset mapped by the randomized algorithm. From Formula (6), two similar datasets are randomized by the function A(x) and the probability ratio of both randomized datasets belonging to same set is equal or lower than exp(ϵ), which can quantitatively indicate the difference between two datasets with randomness. In specific applications, the randomized function A(x) can be represented with a real-valued function, $T_{A}\left(x\right)=f\left(x\right)+Y$, where f(x) is the original query or function and *Y* is the noise added to the results of the query or function, which satisfies the randomness of exp(ϵ). We use Laplace noise satisfying $L(0,\sqrt{2}\lambda )$ and the ϵ can be derived as Δf/λ, where Δf is the sensitivity of the certain dataset and is defined as the $L_{1}$ norm of the dataset, $\Delta f=||f\left(D_{1}\right)-f\left(D_{2}\right)\left|\right|$. Therefore, we can apply noises to the GBDT model following the Laplace distribution to protect the decision trees and their hidden raw data.

However, the performance of the model would be severely impaired if we indiscriminately added the same noises to all the nodes of the GBDT model. For a single decision tree, the components contain two different nodes, namely, splitting nodes and leaf nodes, where splitting nodes are built based on the query of the features and leaf nodes are derived from the labels in the training dataset. Therefore, based on the node type, heterogeneous privacy protection is designed to achieve the trade-off between performance and privacy protection.

Specifically, heterogeneous privacy protection means that the noises with different levels are added into the different kinds of nodes. The labels of the dataset are directly acquired from the original data, which are very sensitive; thus, the leaf nodes demand a strengthened protection. In contrast, the features in the dataset are travel-frequency patterns (refer to Section 4), which are preprocessed through two steps, i.e., the original GPS coordination data are first transformed into coarse grid-level trajectories, then grid-level trajectories are transformed from the time domain to the frequency domain. Thus, the original personal GPS trajectories are mapped into less sensitive visiting frequencies, which are queried to generate the threshold value of the splitting nodes. Therefore, fewer noises can be added into the splitting nodes.

**Figure 3:**
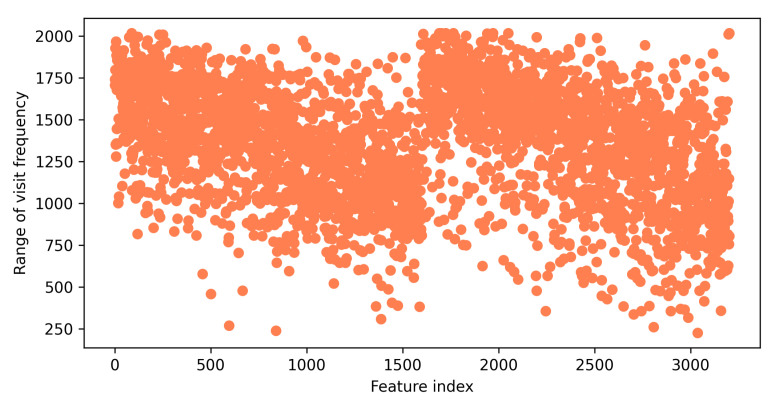
The distribution of travel-frequency patterns in long-term mobility data.

Moreover, referring to Figure 3, the statistics of feature distribution in the long-term mobility dataset vary a lot, from 250 to 2000 on each grid. Moreover, the feature dimension is 3202, which means that the number of possible values of splitting nodes is very large, over 4 million (feature dimension, 3202 *; then, average of visiting frequency on each grid, 1346 = 4,309,892). The max number of splitting nodes and leaf nodes in each decision tree can be derived from the formula $n_{splitting\_nodes}=\left(2^{d-1}-1\right)$ and $n_{leaf\_nodes}=2^{d-1}$, where *d* is the max depth of the decision tree. For example, setting the depth of the decision tree as d=7, the number of splitting nodes is 63, which is quite small compared with the query space of over 4 million (nearly 0.0014%). Oppositely, the labels have only two possible values (1—visited; 0—not visited), which shows that, with splitting nodes, it is harder to expose privacy information than with leaf nodes.

Therefore, we add ϵ noises into the leaf nodes and (h∗ϵ) noises into the splitting nodes in the decision trees, where *h* is a constant (h≥1) to indicate the heterogeneity of noises. The splitting nodes are essential for the performance of each decision tree, which can be less impacted by heterogeneous privacy protection. By setting a proper heterogeneity constant *h*, a better compromise between model performance and privacy protection can be achieved.

### 5.3. FedVoting Process to Construct Federated GBDT Model

Following the analysis in the previous subsection, we add heterogeneous noises to the leaf nodes and splitting nodes of decision trees in the GBDT model, which can prevent the privacy leakage from tree parameters, also having a lesser impact on the performance. Moreover, to protect raw data, the GBDT model is trained on the local position of each silo without the transferring of any original data and derivatives. However, by only implementing this procedure, other silos’ data would not be utilized in the training of the current silo and the performance of the final model would be impaired. Therefore, we propose the FedVoting architecture to construct the model based on the cooperation of different silos, which includes three steps, namely, training, cross-validation and voting.

**Figure 4:**
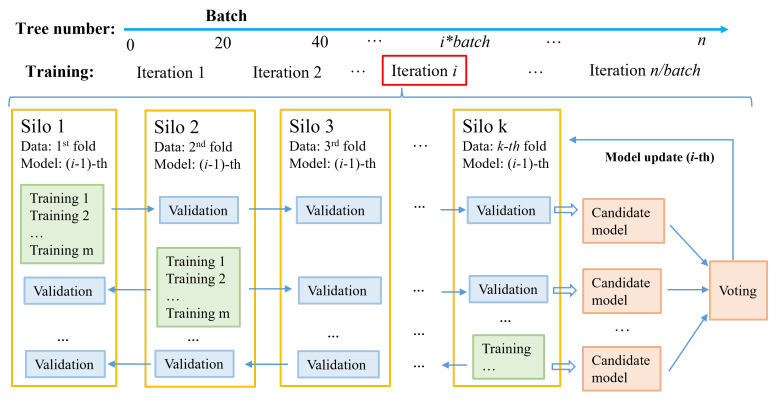
The construction process of FedVoting.

As Figure 4 shows, we suppose there are *k* silos, which can be viewed as the total dataset divided into *k* folds, with each silo owning one fold of data. Further, the silos keep their data in the local place and only exchange the trained model and validation results with the server or other silos. The ensemble of the GBDT model contains *n* decision trees and the number of one batch of decision trees is generated in each iteration. Therefore, in iteration *i*, all silos own the (i−1)th model trained in the last iteration (in the first iteration, the (i−1)th model is null). In Figure 4, we use the green-, blue- and orange-filled squares separately to show the structure and process flows of training, cross-validation and voting stages.

In the FedVoting process for one iteration, the first and second steps (training and validation) are similar to K-fold cross-validation with a reverse order. Generally speaking, in traditional K-fold cross-validation, the (k−1) folds of data are utilized for training while one fold of data serve as validation data. This training is repeated *K* times so that each fold of data can serve as the validation role for sufficient model evaluation. However, in the process of FedVoting, there is one silo (fold) training the model, while the other (k−1) silos (folds) serve as the validation roles; furthermore, each silo has an equal chance to train the model. Thus, in this way, private data can be kept in the local position and validation data are more sufficient for the cross validation procedures. After the training stage, the model trained by each silo is transmitted to all other silos and is validated in these silos with their local data. In the voting stage, the model trained by one silo owns (k−1) validated errors, from which the average validation error of each model is calculated, serving as vote. Then, the model with the smallest error is voted as the chosen model in the current iteration to be broadcast to all other silos, called model update. In each iteration, a certain number of trees (one batch) is trained continuously to promote the construction efficiency of the model ensemble. After specific iterations, the whole ensemble of GBDTs is implemented.

Therefore, the federated GBDT model can be constructed by the federated voting process (FedVoting) to achieve long-term human mobility prediction, which can keep these sensitive data in local positions and protect the data privacy of users. Next, we introduce these three steps in a detailed way.

(1) Training stage

The first stage of the FedVoting process is to train the decision trees in the GBDT model with DP protection. As introduced in Section 5.1, the classification model of GBDTs is utilized to achieve long-term human mobility prediction, namely, whether or not the user would attend the special event or visit POIs in the next month based on the travel-frequency patterns of the current month.

The total decision trees in the ensemble of GBDTs are trained by many iterations (*N*). In one iteration, the number of decision trees to be trained is called batch (*b*). Therefore, the tree number of the whole GBDT is the product of iteration number and batch (N∗b). The batch should be equal or over 1; setting a bigger batch can improve the efficiency of training. As Figure 4 shows, each silo participating in federated learning owns its local data and the current models trained by the former (i−1) iterations. When the training stage of the current iteration begins, each participating silo trains a fixed number (batch) of decision trees in a boosted way. Moreover, the training for each silo repeats *m* times to obtain *m* groups of models, which can avoid the randomness that would disturb the performance of the models. Then, the model with the best performance (i.e., with the smallest training error on local data) is chosen as the candidate for the later cross-validation stage.

The processes to train one decision tree with DP protection are shown in the first part of Algorithm 1. The first step (Algorithm 1, from line 1 to line 4) is similar to the common process of building decision trees, the key of which is to set a proper sampling rate of both features and instances to remove overfitting and achieve the best effect of generalization. The second step (Algorithm 1, from line 5 to line 9) consists of adding Laplace noises into the leaf nodes with budget ϵ, as well as splitting nodes with budget (h∗ϵ). The range of noises is decided by the privacy budget, where the smaller the budget, the larger the noise. Therefore, through adjusting the privacy budget, the noise added to the leaf nodes can be controlled to satisfy the demands of both performance and privacy protection.

(2) Cross validation stage

After the training stage, each silo generates *m* groups of decision trees with the number of batches, but only the group with the smallest training error is transmitted to the other (k−1) silos for cross-validation. Therefore, in the cross-validation stage, each silo validates (k−1) models trained separately by the other (k−1) silos on their local data. Then, the silo transfers or uploads only the validation results to the other silos or to the server without any exposure of the original data and privacy being also well protected.

The detailed flows are shown in Algorithm 1, from line 10 to line 15. Firstly, in the current silo, the group of decision trees with the smallest loss is chosen from all the models generated in the multiple training of the current iteration. Then, these decision trees are transmitted to the other (k−1) silos under the coordination of a central server or one of the participated silos. The validation processes are then conducted on these silos based on their local data. Finally, the validation results of the same model from different silos are gathered for the next voting stage.
**Algorithm 1:** The FedVoting process among silos in one iteration.
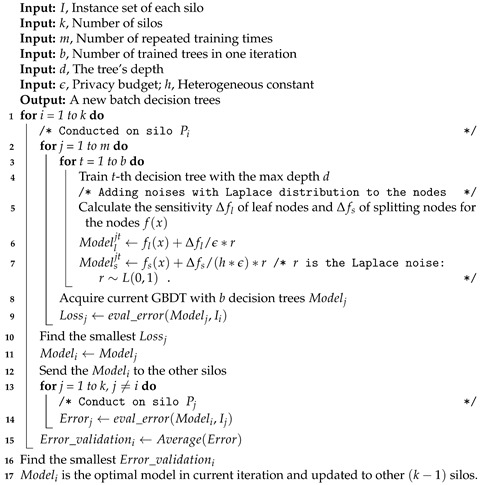


(3) Voting stage

The voting stage is to select the best model for the current iteration. After the validation stage, the model trained by each silo has (k−1) validation results from the data of other silos. Therefore, the validation result can be viewed as the ‘vote’ from other silos for the current model. The ‘vote’ is based on the performance of the model in its local data without exposing the original data, which is reasonable and privacy-preserving. Through selecting the model with the most ‘votes’ (i.e., with the smallest validation error), the batch of decision trees can be decided and put into the final ensemble of GBDTs in the current iteration (Algorithm 1, from line 16 to line 17).

In each iteration, all the silos train the local models with their raw data; then, these models are cross-validated by other silos with totally different raw data. Therefore, the performance of the model trained by one silo can be sufficiently evaluated; the average validation errors of each trained model are calculated and can serve as the votes to facilitate the optimal choice. Finally, the local model with the smallest error is chosen as the optimal model to be aggregated to the GBDT ensemble in the current iteration. Then, the chosen model is updated to all other (k−1) silos.

Overall, after these three stages of one iteration, a batch of decision trees is generated based on the data of all silos, in which one fold of data in one silo serves as the training role and the other (k−1) folds data serve for validating. Through this process, the generated model can avoid over-fitting, compared with using only one silo’s local data. More importantly, FedVoting can effectively protect raw data in a strengthened way, where all individuals’ data and related derivatives are kept in the local position, without being transferred and uploaded. Moreover, for the GBDT models which are transferred or uploaded, we utilize heterogeneous differential privacy to protect the parameters in the decision trees, which also drastically decreases the risk of privacy leakage. Compared with other privacy-preserving methods, such as homomorphic encryption, which has complex encrypting processes and can still be attacked, FedVoting not only protects the data better than other methods, but also has a good performance, which is shown in the next section.

## 6. Experimental Results

In this section, first, we introduce the big GPS dataset and the experiments’ setup. Then, we analyze the training process to show that the FedVoting process is very effective and, as the training goes iteration by iteration, the optimal decision trees are selected and the whole classification errors keep decreasing. After that, we demonstrate the great performance of the FedVoting-constructed federated GBDT model in two long-term human mobility prediction cases (long-term special event attendance and long-term POI visit), compared with the two extreme settings (SOLO and ALLIN, which are introduced later) and the state-of-art baseline. In the end, we elaborate the strengths of the proposed heterogeneous method based on differential privacy in finding a compromise between good performance and privacy protection; further, we show that the FedVoting-constructed federated GBDT model can easily quantify the contributions of each silo to the cooperation, which can facilitate the design of incentive mechanisms and attract more participants in federated learning.

### 6.1. Data

We utilized the “Konzatsu-Tokei (R)” raw GPS log dataset, which refers to people flow data collected by individual location data sent from mobile phones with enabled AUTO-GPS function under users’ consent, through the “docomo map navi” service provided by NTT DOCOMO, INC. These data were processed collectively and statistically in order to conceal private information. Original location data were GPS coordinate data (latitude and longitude) sent in about every 5 min minimum and did not include information to identify individuals, such as gender or age.

In this study, we focused on the GPS dataset within the Greater Tokyo Area over a one-year period (1 January 2012, to 31 December 2012). We divided the Great Tokyo Area into 1 km × 1 km grids and extracted 1600 of the most frequently visited grids as POIs based on the dataset, while the visited grids outside the POIs were considered outliers and set to the same grid index (1601). A user’s trajectories of a specific month were preprocessed to generate travel-frequency pattern data as features in the training and test datasets. In this process, the users with trajectories shorter than 15 days were omitted to improve data quality. Then, we set different special events or POIs as the goal of long-term human mobility prediction, namely, whether the users would have attended the special event or visited the specific public area in the next month.

For special events, Comiket is a very popular activity mainly focusing on the sale of doujin (self-published) works, which is normally held in Tokyo Big Sight twice a year. The C82 Comiket was held from 10 August to 12 August 2012; we extracted the attendees’ information from the trajectories to acquire the labels of users. As shown in Table 2, there were 1965 people who attended the C82 Comiket (positive instances) and 77,952 people who did not attend (negative instances) from the objective users in the dataset. Compared with the number of whole objective users, the users who attended Comiket occupied a small portion, which would have led to severe bias in the prediction results. Therefore, it is necessary to randomly extract the equal portion of negative instances and positive instances to form the final dataset. The numbers in parentheses of Table 2 are the quantities used in the dataset. In this research study, we focused on the horizontal federated learning setting and the features of data were the same in all silos. Therefore, the whole dataset could be divided equally or with different portions and then distributed to each silo.

For the prediction of visiting POIs, we chose Disneyland as the research POI. Similarly to Comiket, we extracted the visiting information for the whole month of August, 2012, in the original dataset. Specifically, if a user visited Tokyo Disneyland once or more in the whole of August, he or she was considered a positive instance; otherwise, the user was labeled as a negative instance. In this way, the dataset could be generated and then adjusted to have an equal portion of positive and negative instances.

### 6.2. Experiments’ Setup

We conducted the experiments on a workstation running Linux with Intel Xeon E5-2690v4 CPU (2.6 GHz 14C 35M 9.60 GT/sec 135W), 2× TitanX Pascal 12 GB GDDR5X graphics card, 128 GB (8× 16 GB DDR4-2400 ECC RDIMM) and a 1.2 TB Intel NVme DC P3600 Series SSD. We used 70% of the dataset as training data and the remainder for testing. Then, the training dataset was randomly divided into *k* equal parts as the local data of *k* silos and the max depth of GBDTs was set to 7.

We first demonstrate the training process of the FedVoting architecture. Then, we report the testing of two principal baselines, ALL-IN and SOLO, to compare with the performance of FedVoting. ALL-IN means that the data in all silos were put together to train a model, which represents the best situation where the data can be gathered together without privacy concerns. SOLO means that each silo trained the model by using only their own data, without cooperating with other silos, which represents the original conditions. In addition to these two baselines, we also tested a baseline called SimFL, which is also a federated GBDT model constructed based on the similarities among the instances of different participants [8]. Then, we present the classification errors of FedVoting to compare with these baselines, to show the high performance and practicality in the problems of long-term prediction of POI visits, including both special event attendance and POI visit prediction. Next, we analyze the privacy protection and incentives for participants joining federated learning. To avoid the randomness, we ran FedVoting 10 times to acquire the average results.

For the prediction of long-term human mobility, we used the classification error to state the performance of the model, which equaled to (1−accuracy). The accuracy is shown in Equation (7), where TP is true positive, FP is false positive, TN is true negative and FN is false negative. As we focused on the conditions of a balanced ratio of positive to negative samples, the classification error made it very clear and intuitive to demonstrate the performance of models.

(7) $$Accuracy=\frac{TP+TN}{TP+TN+FP+FN}$$

### 6.3. Analysis of the Training Process

**Figure 5:**
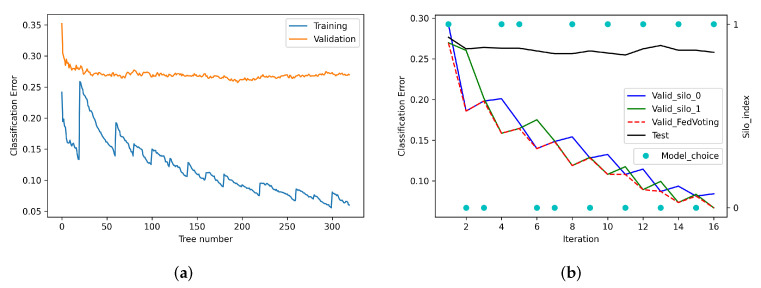
The training process of FedVoting (Silo number = 2, batch = 20). (**a**) The training process in tree numbers. (**b**) The training process in iterations.

As Figure 5a shows, the construction process of the GBDT model based on FedVoting is similar to the normal training pattern. With the increase in tree numbers, the training errors gradually decreased, while the validation errors first decreased and then remained relatively constant. However, we also found that there were some sharp waves in the classification error curve as the tree number increased, which represent the conditions of the training batch and model selection based on the FedVoting process. We refer to Figure 5b for the whole training process (silo number, 2; batch, 20). The blue curve and green curve represent the validation error of the model trained by silo 0 and silo 1, respectively, which is the average value of the validation errors validated by all other silos based on their local data. For example, because there were just two silos, the blue curve of silo 0 is the final validation error of its model, which was validated by silo 1. Based on the final validation error of model, the model with the smallest error is chosen as the final model in the current iteration and is updated to all other silos. For example, in the first iteration, the model (one batch of total 20 trees) trained by silo 1 had the smallest validation error, as the green curve shows. Thus, the model of silo 1 was chosen (shown in cyan-filled circles) and aggregated to the GBDT ensemble, then updated to all other silos. In the second iteration, all silos trained new batches of trees based on the previous model in a addictive way. Then, the model of silo 0 had the smallest validation error, thus, it was chosen as the final model of the iteration and updated to all other silos.

Therefore, after a certain number of iterations, the ensemble of GBDTs could be generated. The training error of the FedVoting process is shown by the dashed red line in Figure 5b, from which we can see that the whole validation errors were low and decreased until the end of the training process. Furthermore, the black line indicates the test error of global test data, showing the generalization performance as the iteration increased. From the above analysis, we can confirm that the FedVoting process could choose the models with the best performance to construct the final ensemble.

### 6.4. Long-Term Prediction of Special Event Attendance

Special events are usually held in a very short period, and some large events can attract tens of thousand people, which is concerning for the government in terms of traffic congestion problems and represents an occasion for companies to promote business activities. Therefore, it is important and beneficial to make attendance prediction in advance. Here, we utilize the GPS dataset in the Great Tokyo Area to predict the attendance of C82 Comiket and show the results.

Table 3 shows the comparison among classification errors to show the performance. There were four participating silos and the data volume was evenly distributed among all silos. For the training of FedVoting, we set the parameter of ϵ in differential privacy to 20 and *h* to 10 (default value, if no extra annotation). We can see that the classification error of the proposed model (FedVoting) was lower than every silo’s training individually (SOLO) and was close to the classification error of ALL-IN (training with all data put together), which means it was beneficial for silos participating in federated learning, so as to acquire the model with the highest performance. Especially, the federated GBDT model constructed with the FedVoting process also had a better performance than the federated GBDT model with similarity (SimFL). Moreover, compared with SimFL, which not only transfers the gradients among participants with the risk of original data leakage but also lacks protection of tree parameters, the decision trees in FedVoting process were trained in local silos without any transferring of raw data or its derivatives. Furthermore, the parameters of the federated GBDT model were protected by differential privacy, which can avoid the privacy leakage and achieve a good performance.

**Figure 6:**
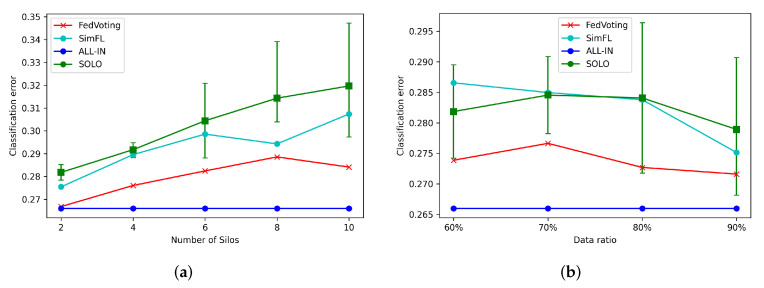
The prediction results of Comiket attendance in different conditions. (**a**) Classification errors of different numbers of silos (Data ratio = 0.5). (**b**) Classification errors of different ratios of the data volume (Silo number = 2).

Next, we analyzed the conditions for different numbers of participating silos to test the effectiveness of the proposed model. As Figure 6a shows, the x-axis represents the number of silos from 2 to 10 and the y-axis represents the classification error. The confidence interval of the SOLO condition (green curve) represents the distribution of the classification errors of the model trained by each silo. We can see that the classification errors of FedVoting were smaller than those of all SOLO conditions and significantly better than the average performance of all SOLO silos. At the same time, they were close to the ALL-IN conditions. Compared with the performance of SimFL, FedVoting also had smaller classification errors in all conditions for different silo numbers.

The data volume of different silos may be not evenly distributed, which may impact the models trained in federated learning. To show the performance of FedVoting in the conditions of unevenly distributed data volume among silos, we tested the performance with different ratios (θ) (from 60% to 90%) on the condition of two participating silos. As Figure 6b shows, the classification errors of FedVoting were far better than the average of SOLO conditions and mostly better than all single SOLO errors. Further, they were also increasingly closer to the ALL-IN condition, with the increase in the ratio. Compared with SimFL model, FedVoting also achieved a better performance.

From the above analyses, we can find that the federated GBDT constructed by the FedVoting process had a good performance both for different silo numbers and varied data ratios. In this way, the attendance of Comiket could be better predicted through the cooperation of different silos at a negligible expense of privacy exposure.

### 6.5. Long-Term Prediction of POI Visit

In long-term human mobility prediction, another common aspect is the POI visit prediction. In this research study, we used Disneyland as the POI to demonstrate the performance of the proposed models. Disneyland is a very popular entertainment place for most people around the world. Tokyo Disneyland, located in Great Tokyo Area, also has many tourists. As introduced in Section 6.1, we utilized the travel-frequency patterns of the current month to predict whether the user would have visited Tokyo Disneyland in the next month. The classification errors are shown in Table 4; it can be seen that the classification error of FedVoting was also better than all the SOLO conditions and SimFL model and, at the same time, close to the ALL-IN condition.

**Figure 7:**
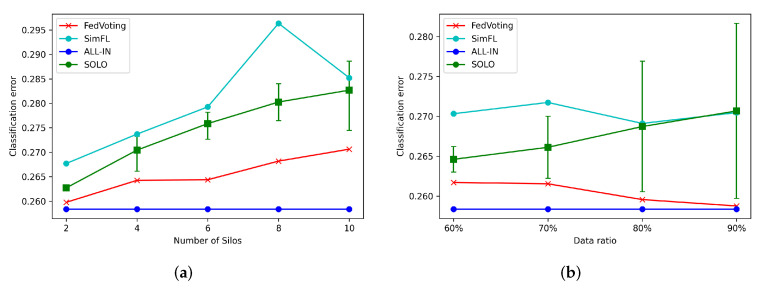
The prediction results of Disneyland visit in different conditions. (**a**) Classification errors of different numbers of silos. (**b**) Classification errors of different ratios of data volume.

Similar to the demonstration of Comiket attendance prediction, the Disneyland visiting predictions are also displayed for different participating silos and different data volume ratios, as shown in Figure 7. The classification errors of FedVoting were better than all single SOLO errors. Further, they were also close to the classification error of the ALL-IN condition. We can see that the SimFL model performed worse than even the SOLO conditions, which indicates that the similarity patterns are not suitable for travel-frequency pattern data; the similarity among different silos cannot contribute to a better performance of prediction but, rather, worsen the prediction accuracy.

### 6.6. Privacy-Protection Analysis

As introduced in the previous sections, the federated GBDT model constructed with the FedVoting process consists of decision trees separately trained by different silos on their local data, which means that the original data in each silo are naturally protected. However, the parameters of the decision tree in the GBDT model still contain some private information, because these parameters are queried from the original data in the silos. To further protect privacy, we analyzed the risk of privacy leakage for the different kinds of tree parameters (splitting nodes and leaf nodes) and designed the heterogeneous privacy protection method to prevent privacy leakage as well as improving the performance of the model.

**Figure 8:**
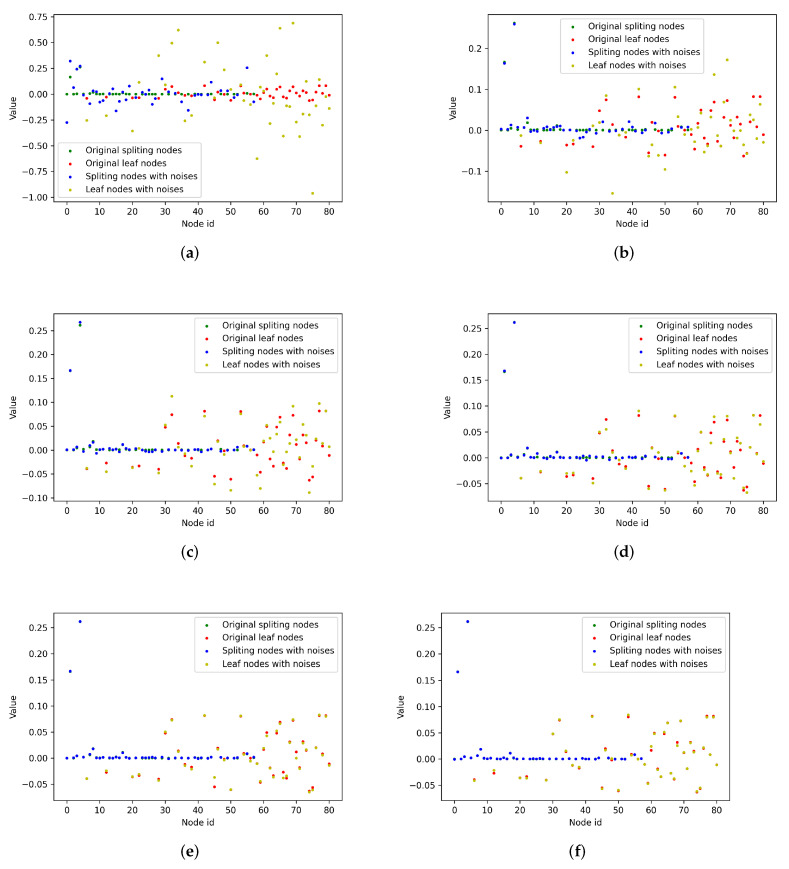
The noises added to the leaf nodes for different ϵ (h=10). (**a**) ϵ=0.5. (**b**) ϵ=5. (**c**) ϵ=10. (**d**) ϵ=20. (**e**) ϵ=50. (**f**) ϵ=100.

In differential privacy, ϵ (epsilon) denotes the amount of noise added. The smaller the ϵ, the larger the noise added. In heterogeneous privacy protection, ϵ-Laplace noises are added to the leaf nodes and (h∗ϵ)-Laplace noises are added to the splitting nodes. When *h* is fixed (e.g., h=20), the noises level can be adjusted by selecting different values of ϵ. To further balance the performance and privacy protection, the appropriate ϵ should be decided. As shown in Figure 8, for each graph, the green points represent the original values of the splitting nodes and the blue points show the values with different Laplace noises. Similar to leaf nodes, the red points are the original values and the yellow points are the values with different ϵ-noises. We can see that the noises were significantly larger than then the original values in the condition with ϵ=0.5 (Figure 8a), which would lead to terrible prediction accuracy, even though privacy would be well protected. Similarly, when ϵ=50 or ϵ=100 (Figure 8e,f), the noises added were too small to realize privacy protection, although the performance of the model would be less impacted. However, in the condition with ϵ chosen as 5, 10 and 20, the noises were relatively intermediate; therefore, they would be able to achieve a good performance.

**Figure 9:**
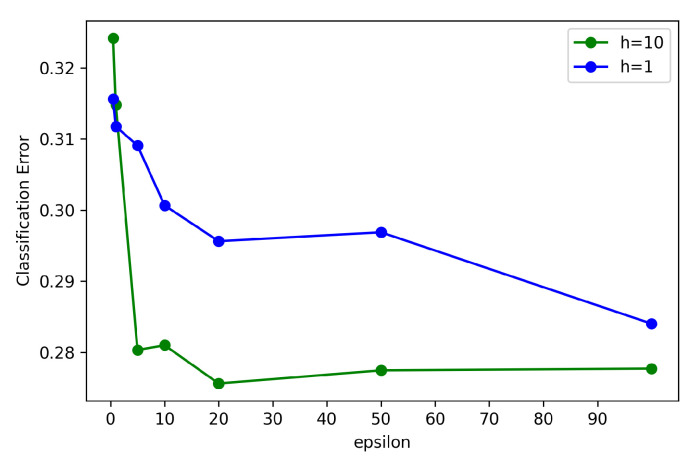
The classification errors for different ϵ(silonumber=4).

After confirming the condition of the noises added into the model for different values of ϵ, we tested the real accuracy of long-term prediction of POI visit based on the Comiket dataset, which is shown in Figure 9. The blue line shows the condition for h=1, which means the levels of added noise were the same in both the leaf nodes and splitting nodes. We can see that the classification errors were very large, regardless of the ϵ chosen. Differently, in the conditions of heterogeneous privacy protection, the classification error drastically decreased with the increase in ϵ, shown as the green line (h=10). Especially, with ϵ=20, the classification errors were pretty small, which would achieve a compromise between performance and privacy protection.

Overall, the FedVoting process chooses the DP-protected decision trees with the smallest validation error into the final ensemble of federated GBDTs in each iteration. The degree of privacy protection can be set by the parameter ϵ with the heterogeneous method. For different levels of privacy protection, FedVoting can acquire the model with the best performance. In common cases, we should choose the intermediate value of ϵ to achieve a good balance between performance and privacy protection.

### 6.7. Motivation Mechanism Analysis

In federated learning, many participants cooperate together to train a collective model based on their local data. However, the premise of cooperation is that there are enough motivations for silos to be willing to join in. As introduced above, the most important motivation for which all silos can acquire a collective model with better performance than models trained by a single silo and at the same time is that the original data of each silo can be kept locally without uploading or transferring to protect user privacy. However, this cannot guarantee fairness, because all silos obtain the same benefits (high-performance model), while the contributions to model training may be varied. Therefore, we need to explore the mechanism of incentives to guarantee fairness and attract silos to join federated learning.

**Figure 10:**
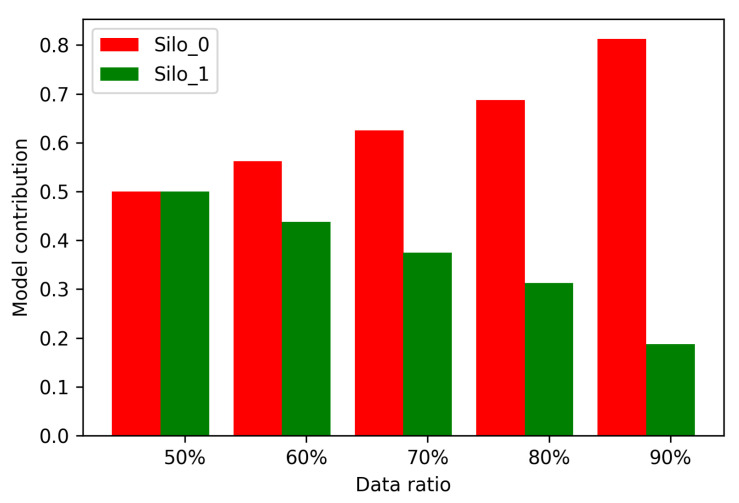
The proportion of model contribution of silos in different data volumes.

Firstly, we should acquire the specific contributions of different silos in federated GBDTs. The FedVoting process supplies a very objective indicator of the contribution. In each iteration, the FedVoting process selects a batch of decision trees with the best performance to put into the final ensemble of GBDTs. This batch of decision trees is trained by a specific silo. After finishing all the iterations, the trees in the final ensemble can be counted from the suppliers (silos). The larger the number of trees trained by a silo, the greater the contribution of this silo. Therefore, we acquire the indicator of the contribution of each silo, which is the proportion of the decision trees trained by each silo in the final ensemble. As Figure 10 shows, there are two silos (silo 0 and silo 1) and the ratio of data volume among them varies (from 50% to 90%). When the data volumes are equal, the model contribution is same. As the ratio of data volume in silo 0 increases, the model contribution also increases and the contribution of silo 1 decreases.

Therefore, based on the quantilized indicator of model contribution for each silo, there are some incentives that can be designed to achieve fairness and attract participants. For example, the silo with low contribution can pay money to the silo with high contribution; this is beneficial for both silos, because the silo with low contribution would acquire a much better model and the silo with high contribution would obtain some payback with money.

## 7. Conclusions and Future Work

Long-term human mobility predictions are irregular and difficult to make. However, we can first extract rich features from the GPS trajectories of users to generate travel-frequency patterns as the input; then, we can choose GBDTs as the base model to conduct the classification, because GBDTs, as a tree model, can better tackle the high-dimensional features and achieve a good performance compared with other methods, such as matrix factorization, deep neural network, etc. Considering the privacy protection of local data, we apply federated learning to the construction of the GBDT ensemble and propose the FedVoting process to conduct optimal model selection in the iterations of the training process. At the same time, to further protect user privacy, we design the heterogeneous method to separately protect the leaf nodes and splitting nodes in the decision trees based on differential privacy, which can achieve a good compromise between performance and privacy protection. Finally, we tested the proposed model in two kinds of long-term human mobility prediction scenarios (special event attendance and POI visit) and conducted the comparison with the baselines of ALL-IN, SOLO and start-of-art prediction models. As shown in the figures in the previous section, the classification errors of FedVoting were all better than the SOLO conditions and the SimFL model and very close to the ideal ALL-IN conditions without privacy concerns. From the privacy analyses, we can see that, as the ϵ became smaller (namely, more noises were added into the model), the classification errors increased rapidly. However, with the proposed heterogeneous method based on differential privacy, the classification errors were smaller in the same ϵ, which achieved a good compromise between performance and privacy protection. Furthermore, the federated GBDT model trained by the proposed FedVoting process could easily provide the quantitative contributions for each participated silos, which can facilitate the design of incentives to attract silos to join federated learning.

For further exploration, in the aspect of practicability, we believe that more conditions for different participating silos could be considered, such as one silo having more GPS data in a certain region or more accurate GPS resolutions. The dataset would be non-independent and identically distributed; utilizing the federated learning method would achieve good performance. Combined with the FedVoting process, we can try to optimize the tree structure based on all silo’s data with tolerable privacy leakage. Furthermore, some effective strategies can be tested and combined with the federated GBDT model for a better performance of long-term human mobility prediction, such as context prediction [40,41], similarity analysis of history trajectories [42] and ensemble methods [43].

## Figures and Tables

**Table 1:** The accuracy test of different base models.

Base Model	GBDT	Random Forest	SVM (Linear)	SVM (Sigmoid)	MLP	FM
Accuracy	**0.7305**	0.7195	0.65462	0.62269	0.60705	0.6397

**Table 2:** Dataset overview.

Event	Date	Number of Positive Instances	Number of Negative Instances
Attending Comiket	8.10∼8.12, 2012	1965	77,952 (2000)
Visiting Disneyland	Total August, 2012	9680	80,850 (10,000)

**Table 3:** The comparison of classification errors in Comiket attendance prediction.

Model		Classification Errors		
		Min	Average	Max
ALL_IN		0.26387	0.2695	0.27815
SOLO	Silo 1	0.28571	0.29311	0.30672
	Silo 2	0.28487	0.29478	0.30588
	Silo 3	0.28319	0.28823	0.29495
	Silo 4	0.27647	0.29084	0.29916
**FedVoting**		**0.26387**	**0.27605**	**0.28571**
SimFL		0.27647	0.28958	0.3

**Table 4:** Comparison of classification errors in Disneyland visit prediction.

Model		Classification Errors		
		Min	Average	Max
ALL_IN		0.25559	0.25837	0.26118
SOLO	Silo 1	0.27473	0.27680	0.27981
	Silo 2	0.26762	0.27265	0.27558
	Silo 3	0.27236	0.27476	0.27642
	Silo 4	0.27473	0.27798	0.28083
	Silo 5	0.27575	0.27818	0.28167
	Silo 6	0.271	0.27476	0.27727
FedVoting		**0.26236**	**0.26438**	**0.26634**
SimFL		0.27575	0.27927	0.28421
